# Adipocyte-derived exosomes from obstructive sleep apnoea rats aggravate MASLD by TCONS_00039830/miR-455-3p/Smad2 axis

**DOI:** 10.1038/s42003-024-06171-z

**Published:** 2024-04-23

**Authors:** Li Yang, Yan He, Shijie Liu, Lulu Gan, Qing Ni, Anni Dai, Changhuan Mu, Qian Liu, Hongyan Chen, Hongying Lu, Ruixue Sun

**Affiliations:** 1 Hypertension Center, Yan ‘an Hospital of Kunming, Kunming, China; 2 Kunming Technical Diagnosis and Treatment Center for Refractory Hypertension, Kunming, China

**Keywords:** Biotechnology, Biochemistry

## Abstract

A correlation exists between obstructive sleep apnoea (OSA) and the severity of metabolic dysfunction-associated steatotic liver disease (MASLD), OSA can induce more severe MASLD. However, the underlying regulatory mechanism between the two is unclear. To this end, this study explored the role and possible molecular mechanisms of adipocyte-derived exosomes under OSA in aggravating MASLD. Through sequencing technology, miR-455-3p was identified as a co-differentially expressed miRNA between the MASLD + OSA and Control groups and between the MASLD + OSA and MASLD groups. Upregulation of TCONS-00039830 and *Smad2* and downregulation of miR-455-3p in the MASLD and MASLD + OSA groups were validated in vivo and in vitro. TCONS-00039830, as a differentially expressed LncRNA in exosomes found in the sequencing results, transfection notably downregulated miR-455-3p and upregulated *Smad2* in hepatocytes. TCONS_00039830 overexpression increased fat, triglyceride and cholesterol levels, while miR-455-3p overexpression decreased these levels. Furthermore, exosome administration promoted the accumulation of fat, triglyceride and cholesterol, upregulated TCONS_00039830 and *Smad2*, and downregulated miR-455-3p. Overexpression of miR-455-3p reversed the increased fat accumulation and upregulated TCONS_00039830 and *Smad2*. In conclusion, OSA-derived exosomes promoted hepatocyte steatosis by regulating TCONS_00039830/miR-455-3p/*Smad2* axis, thereby aggravating liver damage in MASLD.

## Abstract (web summary)

Exosomes from rats with obstructive sleep apnoea aggravate metabolic dysfunction-associated steatotic liver disease by delivering the lncRNA TCONS_00039830 to hepatocytes and regulating Smad2 expression through targeting miR-455-3p.

## Introduction

Metabolic dysfunction-associated steatotic liver disease (MASLD) is the most common liver disease characterised by non-alcoholic hepatic steatosis caused by factors other than excessive alcohol consumption. It is a progressive condition in which further hepatocyte damage is induced by sustained liver injury. The disease includes a spectrum of disease severity, ranging from steatosis without inflammation to non-alcoholic steatohepatitis and hepatocellular carcinoma or liver cirrhosis. It is an emerging disease with a global prevalence of approximately 25%^1^, and its incidence correspondingly increases with that of metabolic diseases, especially among individuals with obesity^2^. Obstructive sleep apnoea (OSA) is defined as a total loss of respiratory airflow or repetitive episodes of partial obstruction of airflow during sleep accompanied by strenuous breathing and collapse of the upper airway during inspiration^3^. Notably, the severity of OSA has been associated with the progression of MASLD^4^. The findings of a systematic review indicated an increase in the prevalence of MASLD among patients with OSA and suggested that the occurrence of OSA is associated with the symptoms of MASLD, including fibrosis, lobular inflammation and steatosis^5^. Salvatore et al. proposed that treating sleep-disordered breathing could help in the management of patients with severe MASLD^6^. Overall, a correlation may exist between OSA and the severity of MASLD; however, the underlying molecular mechanisms remain unclear.

Exosomes are the small vesicles in many biological fluids^7^ containing multiple functional mRNAs, miRNAs and proteins^8,9^. These extracellular vesicles participate in various physiologic processes, including cell proliferation, metabolism and differentiation^10,11^. Exosomes are widely distributed in the tumour microenvironment and transport bioactive lipids through lipid carriers in their bilayer membrane. They are considered one of the main contributors to tumour progression and metastasis, specifically lipid metabolism^12^. Recently, some authors have reported the role of adipocyte-derived exosomes in the pathogenesis of metabolic disorders, such as insulin resistance^13^, obesity-related liver disease^14^ and liver cancer^15^. Therefore, we speculate that exosomes may mediate the contribution of OSA in the pathogenesis of MASLD.

LncRNAs are involved in multiple cellular processes, including proliferation, differentiation, cell cycle regulation and apoptosis^16^. Bioinformatic analysis has revealed their role in several diseases, such as MASLD. RNA sequencing of liver tissue samples revealed 1735 differentially expressed lncRNAs between patients with MASLD and healthy individuals, indicating their potential critical role in the development and progression of MASLD^16^. Chen et al. identified AK012226 as a lncRNA involved in lipid accumulation in MASLD^17^. UC372 is upregulated in MASLD and can initiate hepatic steatosis by inhibiting the expression of target genes related to miR-195/miR-4668^18^. In this study, sequencing data revealed that TCONS-00039830 is upregulated in MASLD. The expression level of TCONS-00039830 was high in OSA and exosomes derived from rats with OSA. Therefore, we speculated that adipocyte-derived exosomes from rats with OSA initiate hepatic steatosis by activating TCONS_ 00039830, which further accelerates the progression of MASLD.

In this study, we aimed to investigate the mechanisms through which OSA accelerates MASLD. The transcriptomes of liver tissues of Control rats, rats with MASLD and rats with both MASLD and OSA were sequenced. In addition, rat models and MASLD cell models with silenced TCONS_00039830 and overexpressed miR-455-3p/TCONS_00039830 were constructed to determine their role in hepatic steatosis. The relationship among TCONS_00039830, miR-455-3p, and *Smad2* was examined to investigate the involved molecular mechanisms. Our results revealed that exosomes derived from rats with OSA promoted hepatic steatosis to increase the severity of liver damage in MASLD, and the possible molecular mechanism is the increased activity of the TCONS_00039830/miR-455-3p/*Smad2* axis.

## Results

### Differentially expressed mRNAs, lncRNAs and miRNAs

**Fig. 1:**
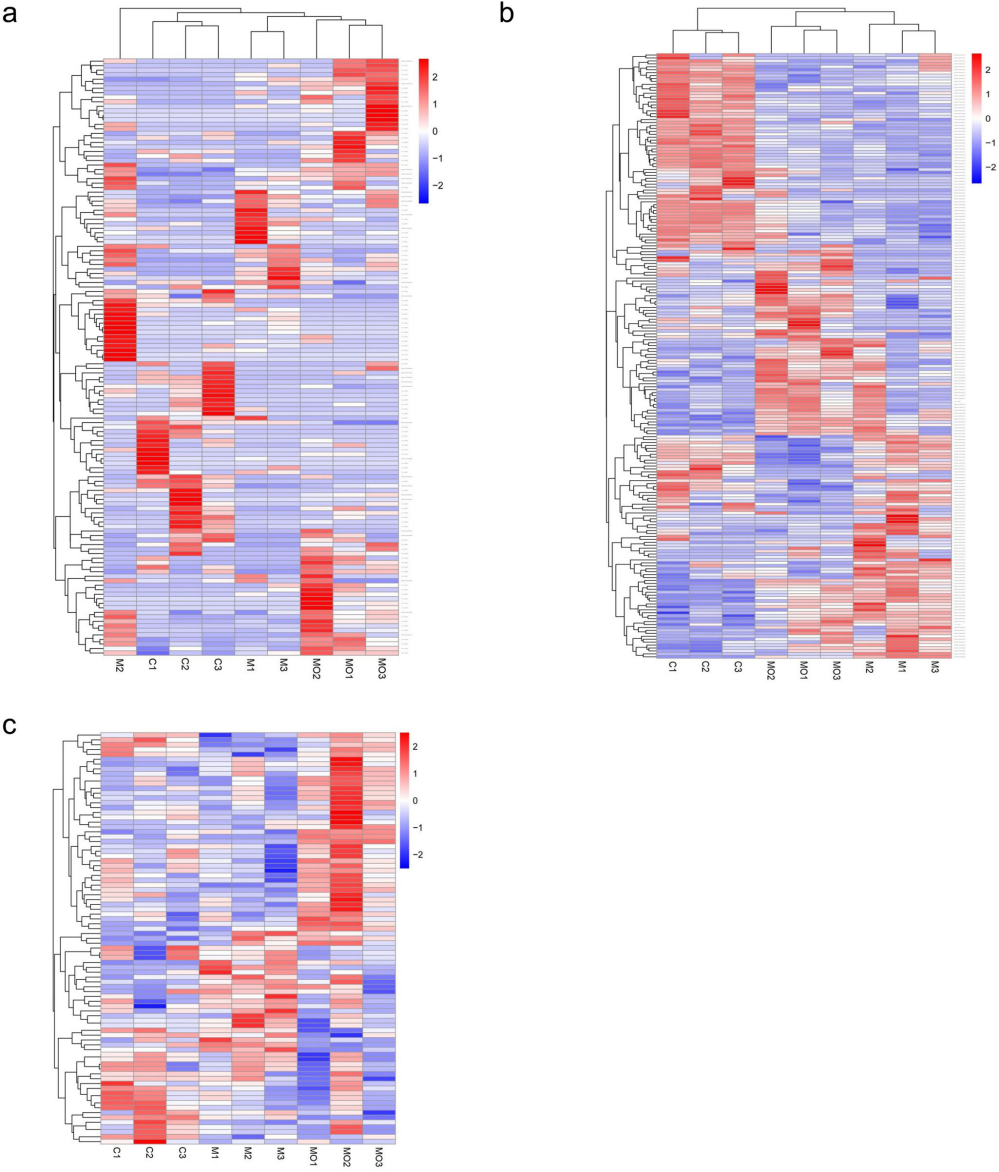
Heatmap of differentially expressed lncRNAs, mRNAs and miRNAs among Control, MASLD, and MASLD + OSA groups. **a** Heatmap of differentially expressed lncRNAs; **b** Heatmap of differentially expressed mRNAs; **c** Heatmap of differentially expressed miRNAs. Blue represents low expression, red represents high expression, and white represents intermediate expression. MASLD Metabolic Dysfunction-associated Steatotic Liver Disease, OSA Obstructive Sleep Apnoea.

Figure 1a, b showed the overall distribution of differentially expressed mRNAs and lncRNAs. Fifty-six differentially expressed lncRNAs (29 downregulated and 27 upregulated; Supplementary Data 1) and 104 differentially expressed mRNAs (58 downregulated and 46 upregulated; Supplementary Data 2) were identified between the MASLD and Control groups. Sixty-six differentially expressed lncRNAs (23 downregulated and 43 upregulated; Supplementary Data 3) and 119 differentially expressed mRNAs (62 downregulated and 57 upregulated; Supplementary Data 4) were identified between the MASLD + OSA and Control groups. Additionally, 44 differentially expressed lncRNAs (19 downregulated and 25 upregulated; Supplementary Data 5) and 61 differentially expressed mRNAs (34 downregulated and 27 upregulated; Supplementary Data 6) were identified between the MASLD + OSA and MASLD groups.

Figure 1c showed the overall distribution of differentially expressed miRNAs. Ten co-differentially expressed miRNAs were identified between the MASLD + OSA and MASLD groups and between the MASLD + OSA and Control groups, including rno-let-7d-5p, rno-miR-106b-3p, rno-miR-10b-5p, rno-miR-152-3p, rno-miR-202-5p, rno, miR-34a-5p, rno-miR-3596b, rno-miR-450a-5p, rno-miR-451-5p and rno-miR-455-3p. Nine co-differentially expressed miRNAs were identified between the MASLD and Control groups and between the MASLD + OSA and Control groups, including rno-miR-182, rno-miR-183-5p, rno-miR-1843a-3p, rno-miR-199a-3p, rno-miR-199a-5p, rno-miR-214-3p, rno-miR-221-5p, rno-miR-3553 and rno-miR-96-5p. Eight co-differentially expressed miRNAs were identified between the MASLD and Control groups and between the MASLD + OSA and MASLD groups, including novel_164, rno-miR-125a-5p, rno-miR-200b-5p, rno-miR-221-3p, rno-miR-3589, rno-miR-425-5p, rno-miR-466b-2-3p and rno-miR-505-3p.

### Functional enrichment of differentially expressed mRNAs

**Fig. 2:**
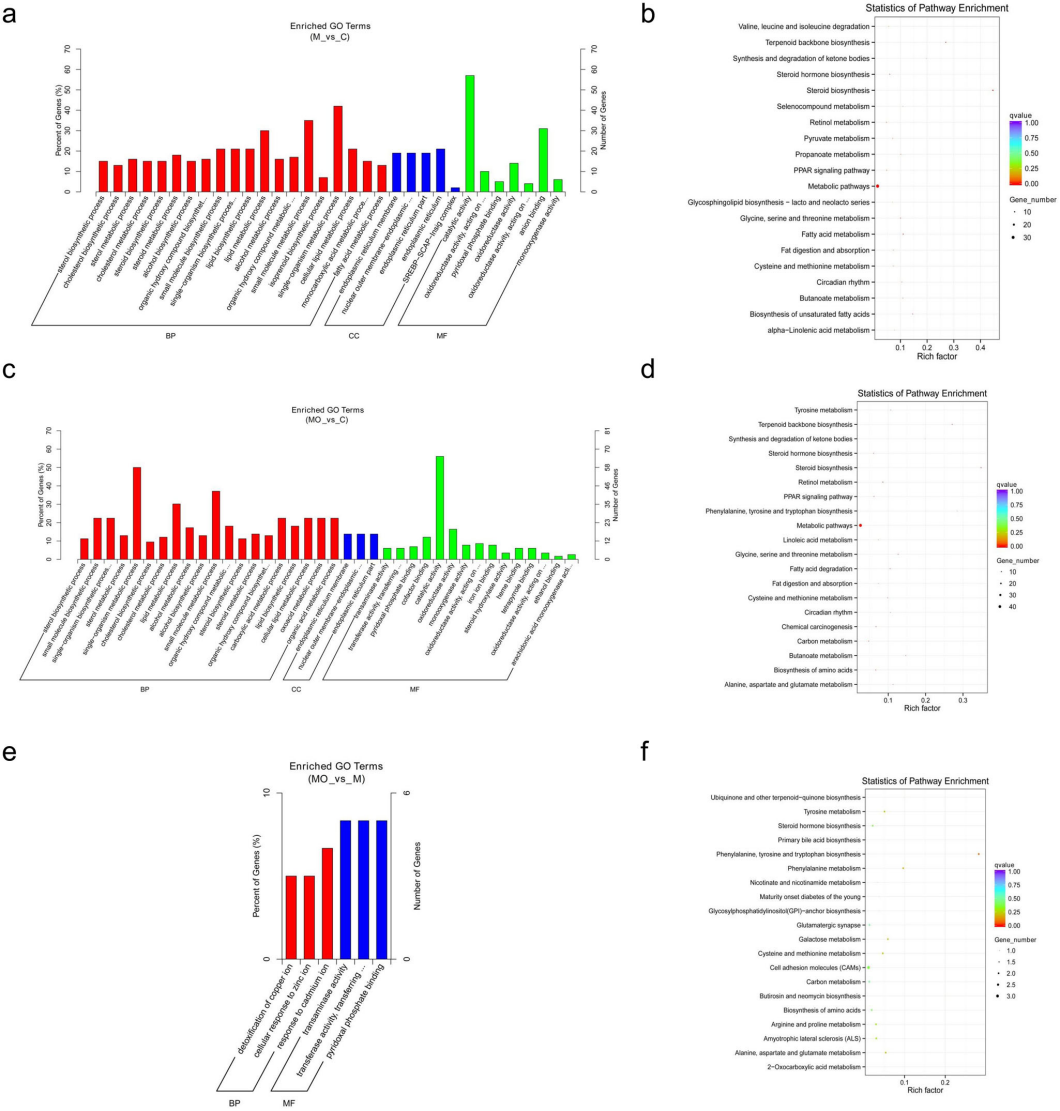
GO and KEGG analyses of differentially expressed mRNAs among Control, MASLD, and MASLD + OSA groups. **a** GO items enriched by differentially expressed mRNAs between the MASLD and Control groups; **b** KEGG pathways enriched by differentially expressed mRNAs between the MASLD and Control groups; **c** GO items enriched by differentially expressed mRNAs between the MASLD + OSA and Control groups; **d** KEGG pathways enriched by differentially expressed mRNAs between the MASLD + OSA and Control groups; **e** GO items enriched by differentially expressed mRNAs between the MASLD + OSA and MASLD groups; **f** KEGG pathways enriched by differentially expressed mRNAs between the MASLD + OSA and MASLD groups. GO Gene Ontology, KEGG Kyoto Encyclopaedia of Genes and Genomes, MASLD Metabolic Dysfunction-associated Steatotic Liver Disease, OSA Obstructive Sleep Apnoea.

KEGG and GO analyses were performed to investigate the functional roles of differentially expressed genes (Fig. 2). The differentially expressed mRNAs in the MASLD group were enriched in biosynthetic and metabolic processes compared with those in the Control group (Fig. 2a). KEGG analysis (Fig. 2b) revealed that differentially expressed mRNAs in the MASLD group were enriched in 16 pathways, including those related to steroid biosynthesis, metabolism, and terpenoid backbone biosynthesis.

Differentially expressed mRNAs between the MASLD + OSA and Control groups were enriched in biosynthetic processes (Fig. 2c) and 18 KEGG pathways (Fig. 2d), including those related to metabolism, steroid biosynthesis, and terpenoid backbone biosynthesis.

Differentially expressed mRNAs between the MASLD + OSA and MASLD groups (Fig. 2e) were enriched in 6 processes, including transaminase activity, transferase activity, transfer of nitrogenous groups, and detoxification of copper ions, and 16 KEGG pathways (Fig. 2f), including those related to phenylalanine, tyrosine and tryptophan biosynthesis, and phenylalanine and galactose metabolism.

### Establishing a lncRNA–miRNA–mRNA network

**Fig. 3:**
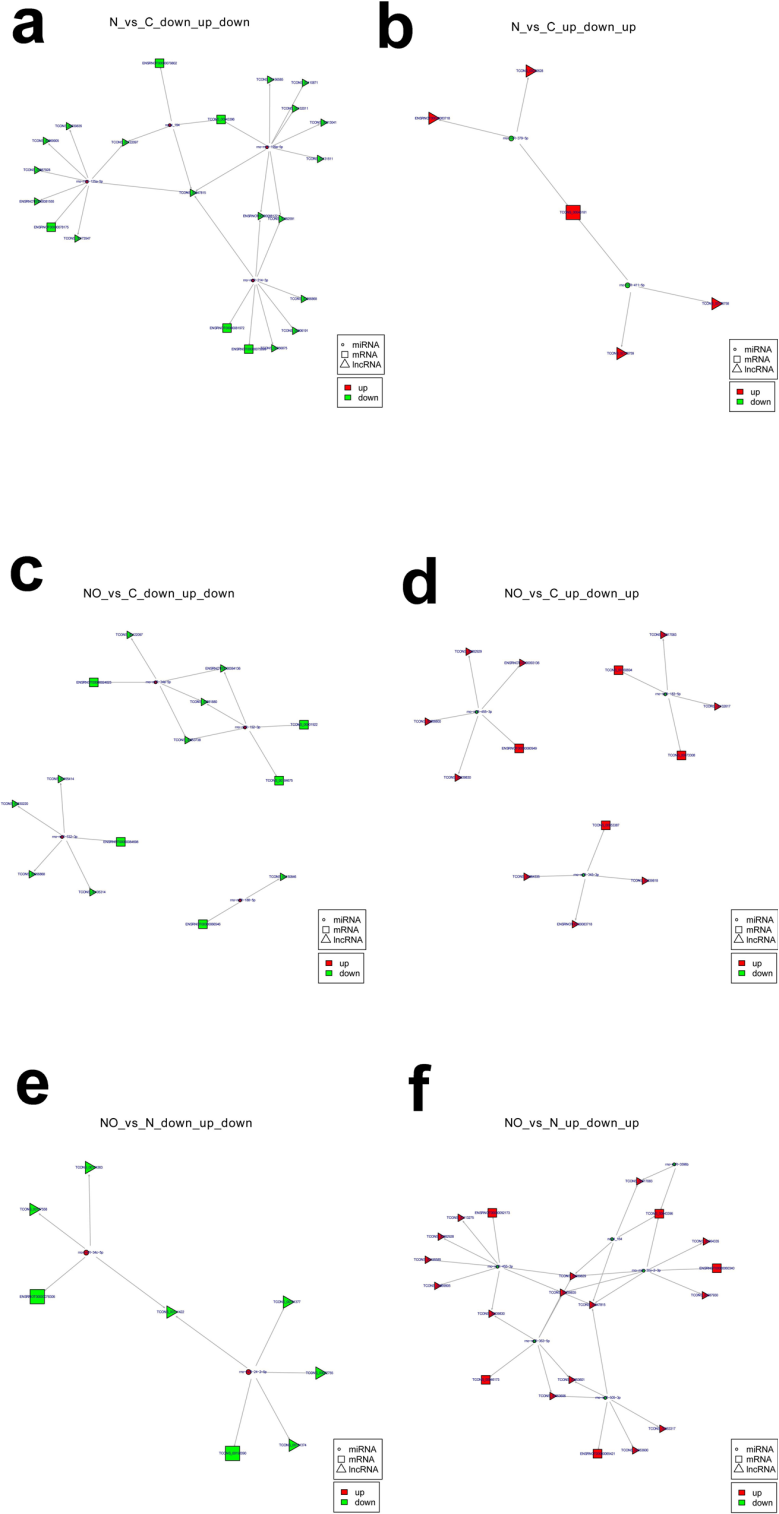
LncRNA–miRNA–mRNA network of differentially expressed genes among Control, MASLD, and MASLD + OSA groups. **a** Downregulated lncRNA–upregulated miRNA–downregulated mRNA network of differentially expressed genes between the MASLD and Control groups; **b** Upregulated lncRNA–downregulated miRNA–upregulated mRNA network of differentially expressed genes between the MASLD and Control groups; **c** Downregulated lncRNA–upregulated miRNA–downregulated mRNA network of differentially expressed genes between the MASLD + OSA and Control groups; **d** Upregulated lncRNA–downregulated miRNA–upregulated mRNA network of differentially expressed genes between the MASLD + OSA and Control groups; **e** Downregulated lncRNA–upregulated miRNA–downregulated mRNA network of differentially expressed genes between the MASLD + OSA and MASLD groups; **f** Upregulated lncRNA–downregulated miRNA–upregulated mRNA network of differentially expressed genes between the MASLD + OSA and MASLD groups. MASLD Metabolic Dysfunction-associated Steatotic Liver Disease, OSA Obstructive Sleep Apnoea.

LncRNAs can act as miRNA sponges by competitively binding to miRNAs, thereby diminishing their regulatory effect on mRNAs. Therefore, we constructed an lncRNA–miRNA–mRNA network to explore the potential role of lncRNAs in MASLD (Fig. 3). We constructed a downregulated lncRNA–upregulated miRNA–downregulated mRNA network and an upregulated lncRNA–downregulated miRNA–upregulated mRNA network based on the interactions among lncRNAs, miRNAs and mRNAs and their expression levels.

The downregulated lncRNA–upregulated miRNA–downregulated mRNA network of differentially expressed genes between the MASLD and Control groups revealed downregulated TCONS_00047815 as the hub gene (Fig. 3a). TCONS_00047815 was mediated by upregulated novel_164, miR-125a-5p, miR-199a-5p and miR-214-3p and downregulated ENSRNOT00000079802, ENSRNOT00000078175, ENSRNOT00000075099, TCONS_00043396 and ENSRNOT00000081972.

The upregulated lncRNA–downregulated miRNA–upregulated mRNA network of differentially expressed genes between the MASLD and Control groups had only one mRNA (TCONS_00046181; Fig. 3b). TCONS_00046181 was regulated by miRNAs including miR-379-5p and miR-411-5p and lncRNAs including ENSRNOT00000083718, TCONS_00020628 and TCONS_00176758.

Three relatively independent networks were involved in the downregulated lncRNA–upregulated miRNA–downregulated mRNA network of differentially expressed genes between the MASLD + OSA and Control groups (Fig. 3c). Bdp1, TCONS_00001922 and TCONS_00184675 were regulated by miR-152-3p and miR-34a-5p and mediated by lncRNAs, namely TCONS_00122097, ENSRNOT00000084136, TCONS_00081880 and TCONS_00053738. The expression of Micu2 was regulated by miR-532-3p and mediated by TCONS_00165414, TCONS_00030220, TCONS_00056868 and TCONS_00105314. The Flnb gene was regulated by miR-188-5p and TCONS_00150846.

Three relatively independent networks were involved in the upregulated lncRNA–downregulated miRNA–upregulated mRNA network of differentially expressed genes between the MASLD + OSA and Control groups (Fig. 3d). Kng1 was regulated by miR-183-5p and lncRNAs, including TCONS_00017083 and TCONS_00132617. The TCONS_00160694 and TCONS_00073308 mRNAs were regulated by miR-345-3p and lncRNAs, including TCONS_00039818, TCONS_00064335, and ENSRNOT00000083718. The TCONS_00073308 and TCONS_00053387 mRNAs were regulated by miR-455-3p and lncRNAs, including ENSRNOT00000093136, TCONS_00006800, TCONS_00039830 and TCONS_00082929.

The downregulated lncRNA–upregulated miRNA–downregulated mRNA network of differentially expressed genes between the MASLD + OSA and MASLD groups showed that downregulated Acap3 was regulated by downregulated lncRNAs, including TCONS_00111422, TCONS_00174363 and TCONS_00107558 and upregulated miR-34c-5p (Fig. 3e).

The upregulated lncRNA–downregulated miRNA–upregulated mRNA network of differentially expressed genes between the MASLD + OSA and MASLD groups included 15 upregulated lncRNAs, 5 downregulated mRNAs and 5 upregulated mRNAs (Fig. 3f). Upregulated *Smad2* was regulated by downregulated TCONS_00039830 and upregulated miR-455-3p in this network.

### Verifying the expression of miR-455-3p, TCONS_00039830 and *Smad2* in rats with MASLD and both MASLD and OSA

**Fig. 4:**
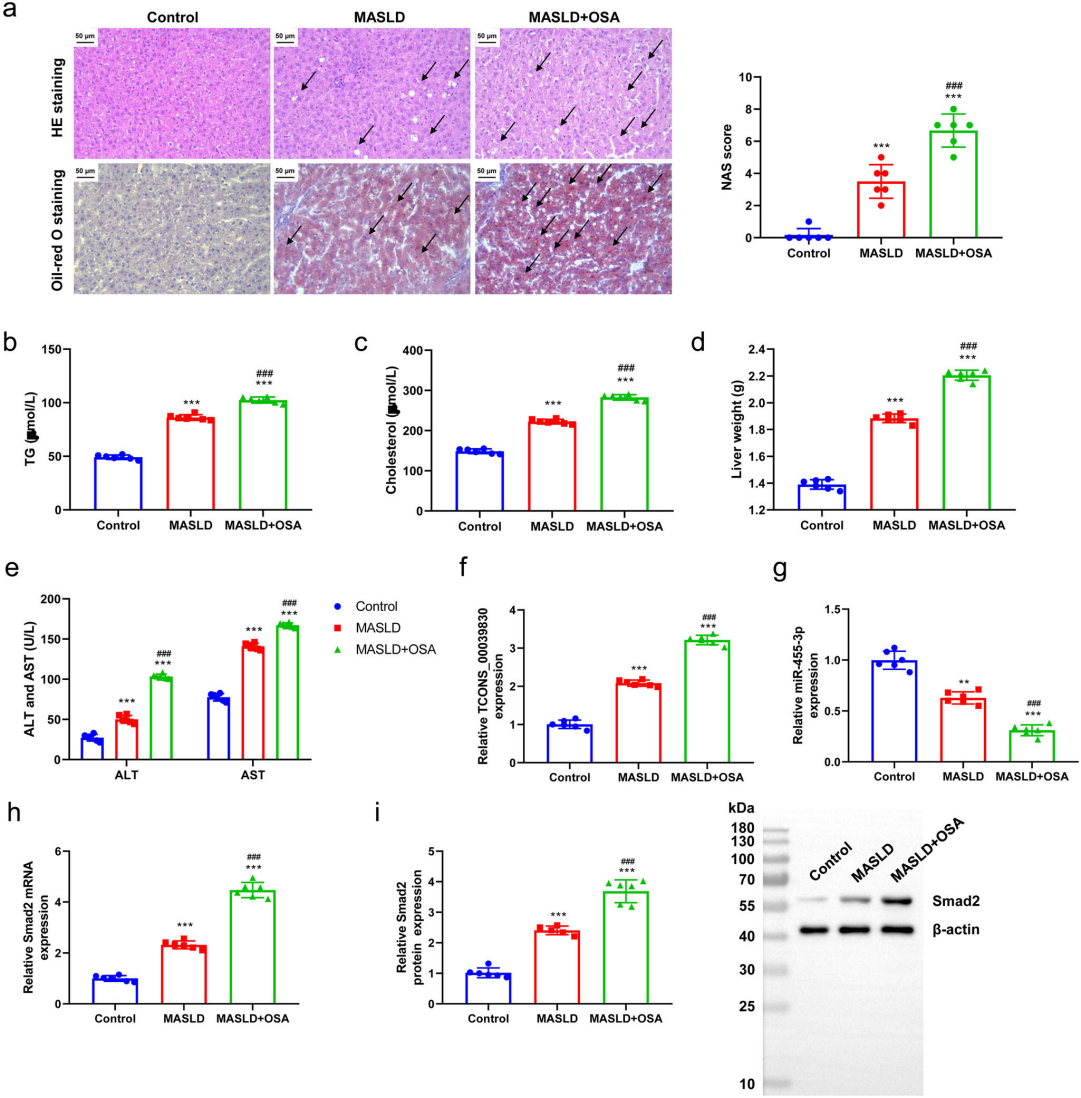
Verifying the expression of miR-455-3p, TCONS_00039830 and *Smad2* in rats with MASLD and both MASLD and OSA. **a** HE staining and oil red O staining of liver tissues of rats in Control, MASLD, and MASLD + OSA groups, as well as NAFLD activity score (NAS); arrows point out histopathological changes; **b** Triglyceride level of rat serum in Control, MASLD, and MASLD + OSA groups; **c** Cholesterol level of rat serum in Control, MASLD, and MASLD + OSA groups; **d** Liver weight of rats in Control, MASLD, and MASLD + OSA groups; **e** Aspartate aminotransferase (ALT) and alanine aminotransferase (AST) levels of rat serum in Control, MASLD, and MASLD + OSA groups; **f** The relative expression of TCONS-00039830 in liver tissues of rats in Control, MASLD, and MASLD + OSA groups detected by RT–qPCR; **g** The relative expression of miR-455-3p in liver tissues of rats in Control, MASLD, and MASLD + OSA groups assessed by RT–qPCR; **h** The relative mRNA expression of *Smad2* in liver tissues of rats in Control, MASLD, and MASLD + OSA groups measured by RT–qPCR; **i** The relative protein expression of Smad2 in liver tissues of rats in Control, MASLD, and MASLD + OSA groups detected by Western blotting, as well as the quantification results. *n* = 6 rats/group; *P < 0.05, **P < 0.01, ***P < 0.001 versus the Control group; ^###^*P* < 0.001 versus the MASLD group; MASLD Metabolic Dysfunction-associated Steatotic Liver Disease, OSA Obstructive Sleep Apnoea. x̅ ± SD.

H&E and oil red O staining images of the Control group showed that hepatocytes were closely and orderly arranged without the presence of red lipid droplets (Fig. 4a). In contrast, hepatocytes were disordered, diffuse red lipid droplets were seen in the cytoplasm, a few small lipid droplets had fused into large lipid droplets and cellular fat became evident in the MASLD group, and more diffuse red lipid droplets were observed in hepatocytes from the MASLD + OSA group. Furthermore, the NAS of the MASLD group ranged from 2 to 5, and the NAS of all tissues in the MASLD + OSA group was more than 6. Moreover, TG and cholesterol concentrations, liver weight, AST, and ALT were determined. We found that the MASLD + OSA group had higher concentrations of TG (Fig. 4b) and cholesterol (Fig. 4c), liver weight (Fig. 4d), ALT and AST (Fig. 4e) compared with MASLD and Control groups. These findings indicated that rat models of MASLD were successfully established, and OSA exacerbated liver injury and fat accumulation in rats with MASLD. Furthermore, the expression of TCONS-00039830, miR-455-3p and *Smad2* was measured. The mRNA expression of TCONS-00039830 was higher in the MASLD (*P* < 0.01) and MASLD + OSA (*P* < 0.001) groups compared with that in the Control group (Fig. 4f). The mRNA expression of miR-455-3p was lower in the MASLD (*P* < 0.05) and MASLD + OSA (*P* < 0.001) groups compared with that in the Control group (Fig. 4g). Moreover, the results of RT–qPCR and western blotting validated the upregulated expression of Smad2 in the MASLD (*P* < 0.001) and MASLD + OSA (*P* < 0.001) groups (Fig. 4h–i).

### Verifying the relationship among TCONS_00039830, miR-455-3p and *Smad2*

**Fig. 5:**
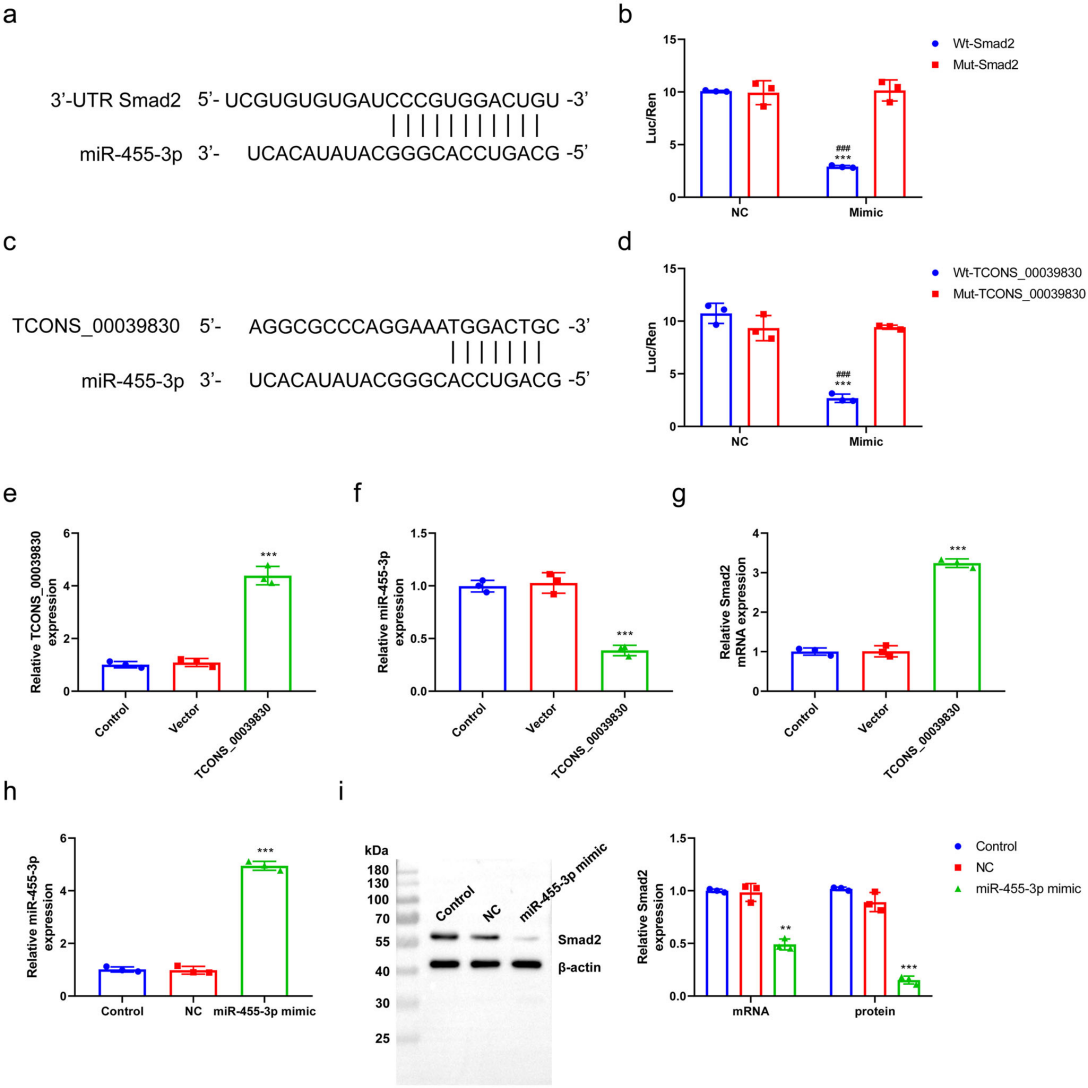
Verifying the relationship among TCONS_00039830, miR-455-3p and *Smad2.* **a** Starbase predicted the binding regions between the *Smad2* 3’-UTR and miR-455-3p; **b** Dual-luciferase reporter assay was performed to verify the targeted binding between miR-455-3p and *Smad2* in hepatocytes; **c** Starbase predicted the binding regions between TCONS_00039830 and miR-455-3p; **d** Dual-luciferase reporter assay was performed to verify the targeted binding between TCONS_00039830 and miR-455-3p in hepatocytes; **e** The relative expression of TCONS_00039830 in hepatocytes transfected with Control, Vector, and TCONS_00039830-overexpression plasmid detected by RT–qPCR; **f** The relative expression of miR-455-3p in hepatocytes transfected with Control, Vector, and TCONS_00039830-overexpression plasmid measured by RT–qPCR; **g** The relative mRNA expression of *Smad2* in hepatocytes transfected with Control, Vector, and TCONS_00039830-overexpression plasmid determined by RT–qPCR; **h** The relative mRNA expression of miR-455-3p in hepatocytes transfected with Control, NC, and miR-455-3p mimic assessed by RT–qPCR; **i** The relative protein expression of Smad2 in hepatocytes transfected with Control, NC, and miR-455-3p mimic measured by Western blotting, as well as the quantification results. **P < 0.01,***P < 0.001, versus NC or Control group; ^###^*P* < 0.001 versus Mut- TCONS_00039830 group. x̅ ± SD.

Dual-luciferase reporter assay was performed to evaluate the relationship among TCONS_00039830, miR-455-3p and *Smad2*. Figure 5a showed the binding between the 3′-UTR of *Smad2* and miR-455-3p. The luciferase activity of wt-*Smad2* was inhibited by miR-455-3p at the 3′-UTR (*P* < 0.001; Fig. 5b), suggesting that miR-455-3p can inhibit the expression of *Smad2* by binding to the 3′-UTR of *Smad2*. Figure 5c shows the binding bases of TCONS-00039830 and miR-455-3p. Finally, miR-455-3p was identified as a downstream target gene of TCONS-00039830 (Fig. 5c, d).

The expression levels of TCONS_00039830, miR-455-3p and *Smad2* were evaluated in cells with TCONS-00039830 overexpression and miR-455-3p overexpression to verify the regulatory relationship among them. Figure 5e, f indicate the successful transfection of TCONS-00039830-overexpression plasmid and miR-455-3p mimic into hepatocytes (*P* < 0.001). MiR-455-3p was downregulated (*P* < 0.001; Fig. 5f) and *Smad2* was upregulated in cells overexpressing TCONS-00039830 (Fig. 5g). Figure 5h indicates the successful transfection of miR-455-3p mimic into hepatocytes (*P* < 0.001). MiR-455-3p was upregulated (*P* < 0.001) and *Smad2* mRNA level (*P* < 0.01) and protein level (*P* < 0.001) was both downregulated in cells overexpressing miR-455-3p (Fig. 5i).

### Role of TCONS-00039830/miR-455-3p/*Smad2* in hepatic steatosis

**Fig. 6:**
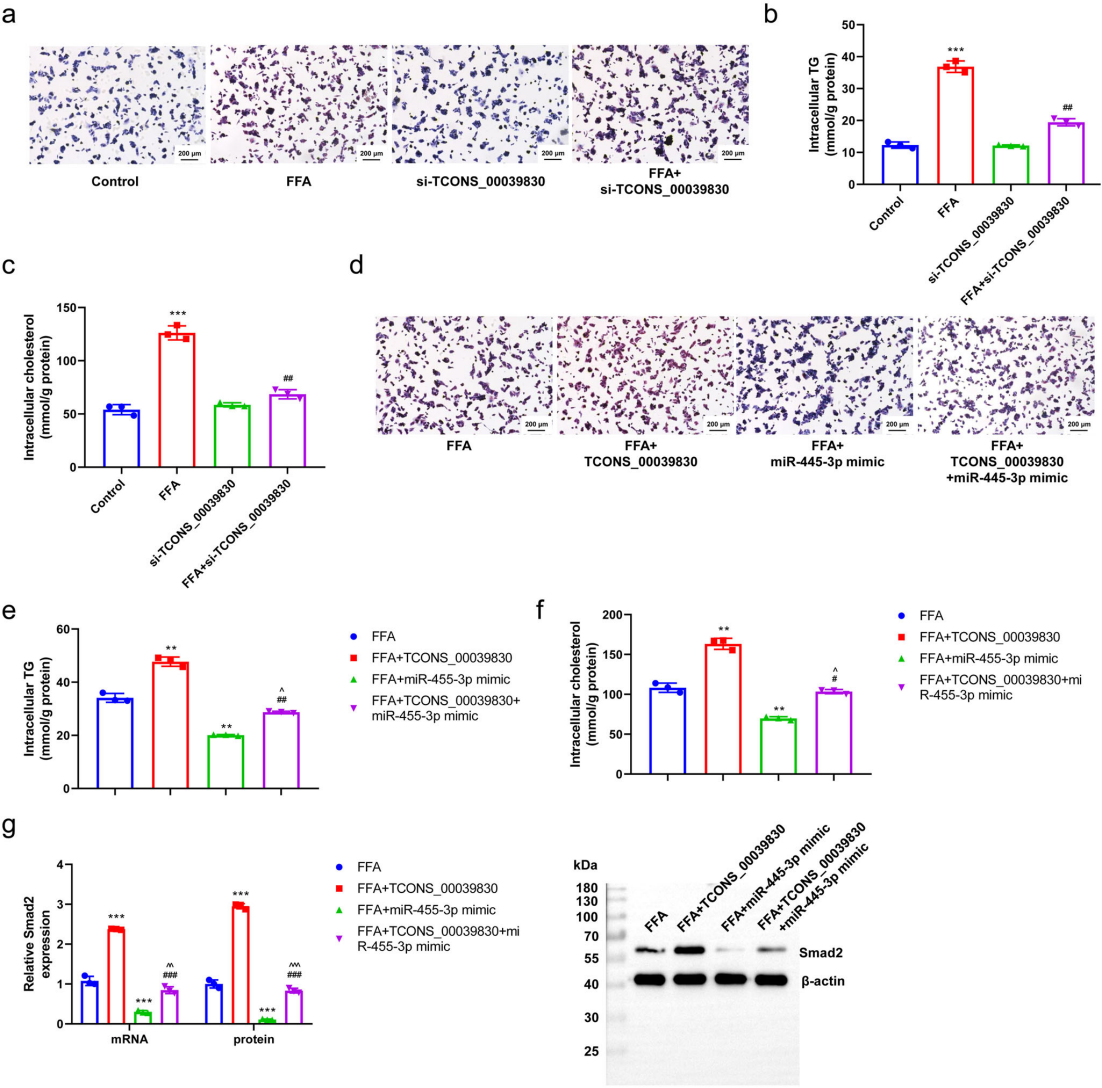
Role of TCONS-00039830/miR-455-3p/*Smad2* in hepatic steatosis. **a** Fat accumulation in hepatocytes in Control, FFA, siTCONS_00039830, and FFA + si-TCONS_00039830 groups detected by oil red O staining; **b** Intracellular triglyceride content in hepatocytes in Control, FFA, siTCONS_00039830, and FFA + si-TCONS_00039830; **c** Intracellular cholesterol content in hepatocytes in Control, FFA, siTCONS_00039830, and FFA + si-TCONS_00039830 groups; **d** Fat accumulation in hepatocytes in FFA, FFA + TCONS_00039830, FFA + miR-455-3p mimic, and FFA + TCONS_00039830 + miR-455-3p mimic groups detected by oil red O staining; **e** Intracellular TG content in hepatocytes in FFA, FFA + TCONS_00039830, FFA + miR-455-3p mimic, and FFA + TCONS_00039830 + miR-455-3p mimic groups; **f** Intracellular cholesterol content in hepatocytes in FFA, FFA + TCONS_00039830, FFA + miR-455-3p mimic, and FFA + TCONS_00039830 + miR-455-3p mimic groups; **g** The relative mRNA and protein expressions of Smad2 in hepatocytes in FFA, FFA + TCONS_00039830, FFA + miR-455-3p mimic, and FFA + TCONS_00039830 + miR-455-3p mimic groups detected by RT-qPCR and Western blotting, as well as the quantification results. *P < 0.05, **P < 0.01, ***P < 0.001 versus Control or FFA group; ^#^*P* < 0.05, ^##^*P* < 0.01, ^###^*P* < 0.001 versus FFA or FFA + TCONS_00039830 group; ^*P* < 0.05, ^^*P* < 0.01 versus FFA + miR-455-3p mimic group; FFA Free Fatty Acid. x̅ ± SD.

Intracellular fat accumulation was evaluated in the FFA + si-TCONS-00039830, FFA + miR-455-3p mimic and FFA + TCONS-00039830 + miR-455-3p mimic groups to examine the role of TCONS-00039830/miR-455-3p/*Smad2* in hepatic steatosis. FFA increased fat accumulation in hepatocytes, whereas TCONS_00039830 silencing reduced FFA-induced fat accumulation (Fig. 6a). Similarly, FFA increased the concentrations of TGs and cholesterol (*P* < 0.001), whereas si-TCONS_00039830 decreased FFA-induced accumulation of TGs and cholesterol (*P* < 0.01; Fig. 6b, c). Furthermore, fat accumulation was compared between cells overexpressing miR-455-3p and those overexpressing TCONS_00039830. Fat accumulation was higher in the FFA + TCONS-00039830 group and lower in the FFA + miR-455-3p mimic group compared with that in the FFA group. MiR-455-3p deceased TCONS-00039830-induced fat accumulation (Fig. 6d). In addition, similar changes were observed in the accumulation of TGs and cholesterol (Fig. 6e, f). The expression of *Smad2* was further evaluated in the three groups to verify its role in the miR-455-3p/TCONS_00039830 pathway. *Smad2* expression was higher in the FFA + TCONS-00039830 group (*P* < 0.001) and lower in the FFA + miR-455-3p mimic group (*P* < 0.01) compared with that in the FFA group. Moreover, miR-455-3p mimic decreased TCONS-00039830-induced upregulation of *Smad2* expression (Fig. 6g). Overall, these results indicated the significance of the TCONS-00039830/miR-455-3p/*Smad2* axis in FFA-induced liver steatosis.

### Exosomes derived from rats with OSA aggravated hepatic steatosis in MASLD

**Fig. 7:**
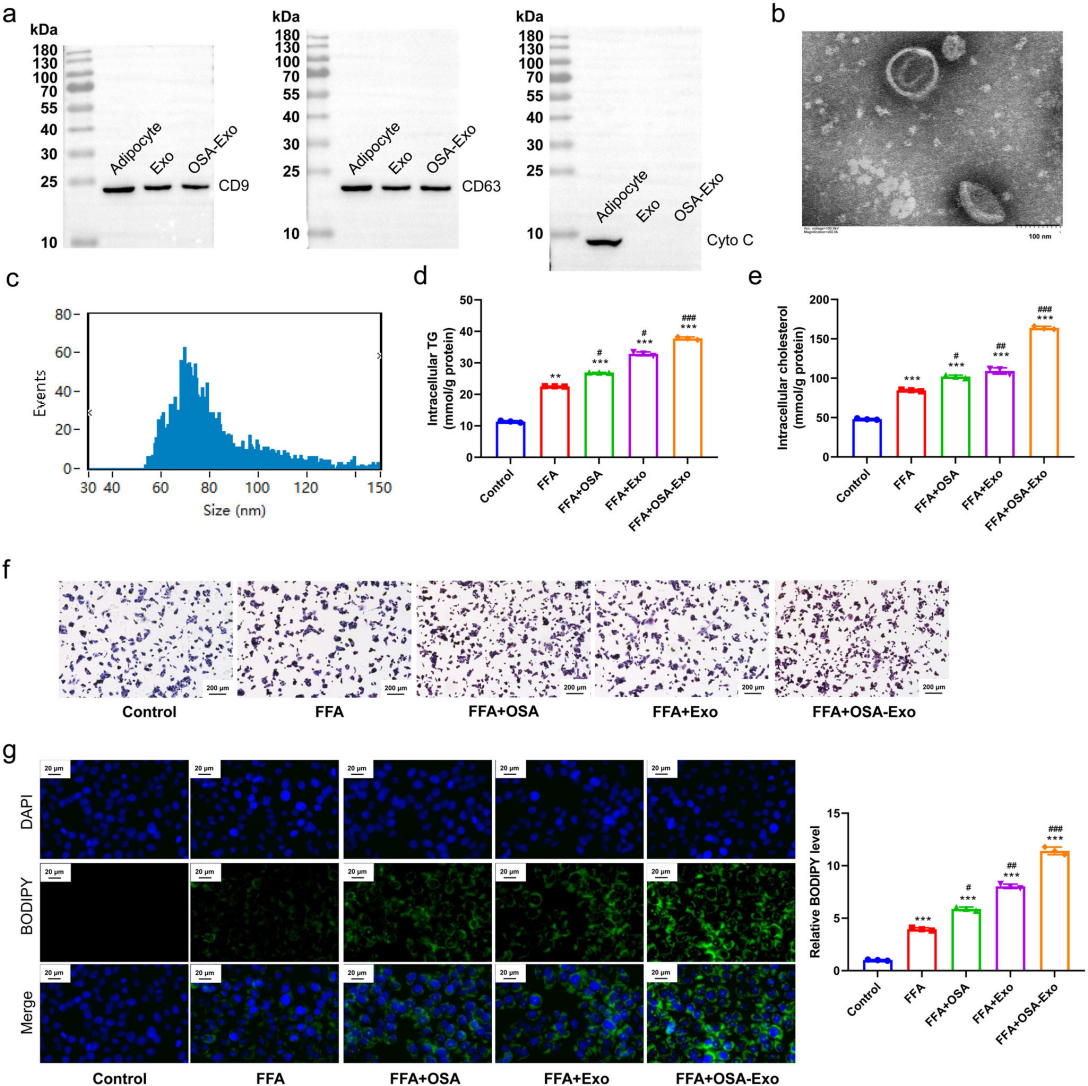
Exosomes derived from rats with OSA aggravates hepatic steatosis in MASLD. **a** The expression of the exosome marker proteins CD9, CD63 and Cyto C in adipocytes, exosomes, and OSA-exosomes detected by Western blotting; **b** Observation of extracted exosomes via transmission electron microscope; **c** The size of exosomes was detected by the particle size analyser; **d** Intracellular triglycerides in hepatocytes in Control, FFA, FFA + OSA, FFA + Exo, FFA + OSA-Exo groups; **e** Intracellular cholesterol in hepatocytes in Control, FFA, FFA + OSA, FFA + Exo, FFA + OSA-Exo groups; **f** Fat accumulation in hepatocytes in Control, FFA, FFA + OSA, FFA + Exo, FFA + OSA-Exo detected by oil red O staining; **g** Hepatic steatosis status in hepatocytes in Control, FFA, FFA + OSA, FFA + Exo, FFA + OSA-Exo groups detected by Bodipy staining, as well as the quantification result. **P < 0.01, ***P < 0.001 versus Control group; ^#^*P* < 0.05, ^##^*P* < 0.01, ^###^*P* < 0.001 versus FFA group; OSA Obstructive Sleep Apnoea; FFA Free Fatty Acid. x̅ ± SD.

Exosomes are important carriers of miRNAs and other biological molecules and play an essential role in understanding cell–cell communication. We isolated serum exosomes from rats with OSA and healthy rats to verify the role of exosomes derived from rats with OSA in promoting hepatic steatosis. Exosomes isolated from rats with OSA expressed CD9 and CD63 but not Cyto C (Fig. 7a). TEM images of the exosomes isolated from the serum indicated their diameter in the range of 60–100 nm (Fig. 7b, c). Triglyceride and cholesterol concentrations were highest in the FFA + OSA-Exo group followed by those in the FFA + Exo, FFA + OSA and FFA groups (Fig. 7d,e). Oil red O staining images revealed that fat accumulation was higher in the FFA + Exo and FFA + OSA-Exo groups compared with that in the FFA and FFA + OSA groups (Fig. 7f). Bodipy staining images indicated that the hepatocytes treated with FFA + OSA-Exo showed the most increased lipid accumulation as compared with FFA + Exo, FFA + OSA, FFA, and Control, and the lipid accumulation of which was higher than that in FFA, and Control (*P* < 0.001; Fig. 7g). Overall, these results suggest that exosomes derived from rats with OSA can lead to fat accumulation in liver tissue and may aggravate hepatic steatosis in MASLD.

### TCONS_00039830 mediated hepatic steatosis induced by miR-455-3p/*Smad2* through exosomes derived from rats with OSA

**Fig. 8:**
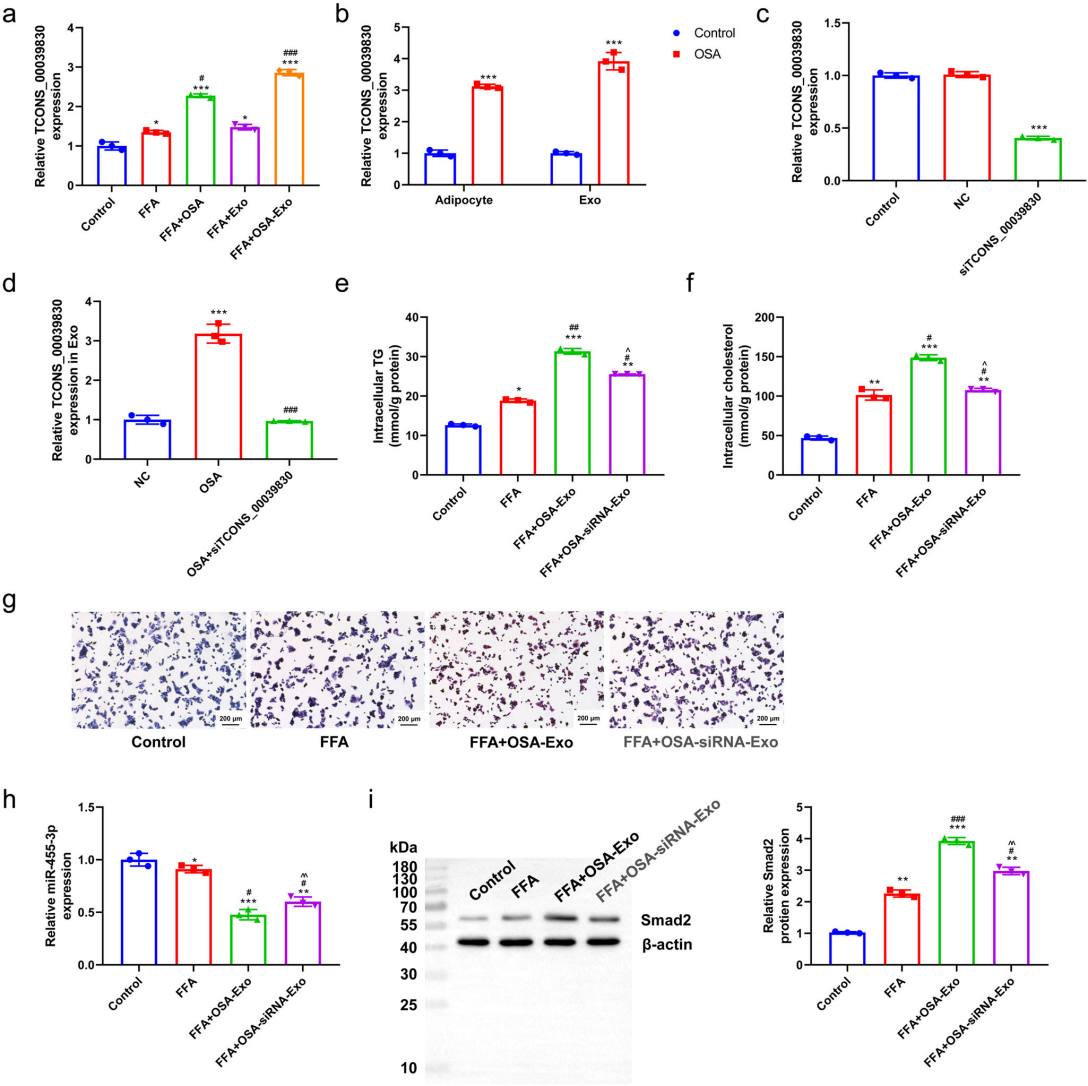
TCONS_00039830 mediates hepatic steatosis induced by miR-455-3p/*Smad2* through exosomes derived from rats with OSA. **a** The relative expression of TCONS_00039830 in hepatocytes in Control, FFA, FFA + OSA, FFA + Exo, and FFA + OSA-Exo groups detected by RT-qPCR; **b** The relative expression of TCONS_00039830 in adipocytes and exosomes from healthy rats and rats with OSA measured by RT-qPCR; **c** The relative expression of TCONS_00039830 in adipocyte-derived exosomes after transfection with si-TCONS_00039830 determined by RT-qPCR; **d** The relative expression of TCONS_00039830 in hepatocytes in Control, OSA and OSA + si-TCONS_00039830 groups assessed by RT-qPCR; **e** Intracellular triglyceride level in hepatocytes in the Control, FFA, FFA + OSA-Exo and FFA + OSA-siRNA-Exo groups; **f** Intracellular cholesterol level in hepatocytes in Control, FFA, FFA + OSA-Exo and FFA + OSA-siRNA-Exo groups; **g** Fat accumulation in hepatocytes detected via oil red O staining; **h** The relative expression of miR-455-3p in hepatocytes in Control, FFA, FFA + OSA-Exo and FFA + OSA-siRNA-Exo groups detected by RT-qPCR; **i** The relative mRNA and protein expressions of Smad2 in hepatocytes from the Control, FFA, FFA + OSA-Exo and FFA + OSA-siRNA-Exo groups assessed by RT-qPCR and Western blotting, as well as the quantification result. *P < 0.05, **P < 0.01, ***P < 0.001 versus Control group; ^#^*P* < 0.05, ^##^ *P* < 0.01, ^###^*P* < 0.001 versus FFA group; ^*P* < 0.05, ^^*P* < 0.01 versus FFA + OSA-Exo group; OSA Obstructive Sleep Apnoea, FFA Free Fatty Acid. x̅ ± SD.

The miR-455-3p/*Smad2* expression and fat accumulation were evaluated in cells with silenced TCONS_00039830 to investigate the mechanism underlying the role of exosomes derived from rats with OSA in promoting fat accumulation. The results of RT–qPCR revealed that TCONS-00039830 expression was highest in the FFA + OSA-Exo group followed by that in the FFA + OSA, FFA + Exo, FFA and Control groups (Fig. 8a). TCONS-00039830 expression in adipocytes and exosomes was compared between Control rats and rats with OSA to examine the role of exosomes in OSA. TCONS-00039830 expression was higher in both adipocytes and exosomes in rats with OSA compared with that in Control rats (*P* < 0.001; Fig. 8b). Furthermore, si-TCONS-00039830 transfection decreased the expression of TCONS-00039830 (Fig. 8c) and OSA-induced upregulation of TCONS-00039830 (*P* < 0.001; Fig. 8d). Treatment with siRNA-Exo decreased the FFA- and OSA-induced increase in TG, cholesterol concentrations (Fig. 8e–f) and fat accumulation (Fig. 8g). Therefore, TCONS-00039830 plays a critical role in modulating fat accumulation in MASLD, and exosomes may act as a mediator in the process. The expression of miR-455-3p was decreased and that of *Smad2* was increased in the FFA + OSA-Exo group. In contrast, si-TCONS_00039830 transfection reversed FFA and OSA-Exo-induced downregulation of miR-455-3p and upregulation of *Smad2* (Fig. 8h–i). Overall, these results suggest that miR-455-3p and *Smad2* are the potential targets of TCONS_00039830 during the modulation of OSA-induced fat accumulation in MASLD.

### Exosome-delivered TCONS_00039830 regulated fat accumulation through miR-455-3p

**Fig. 9:**
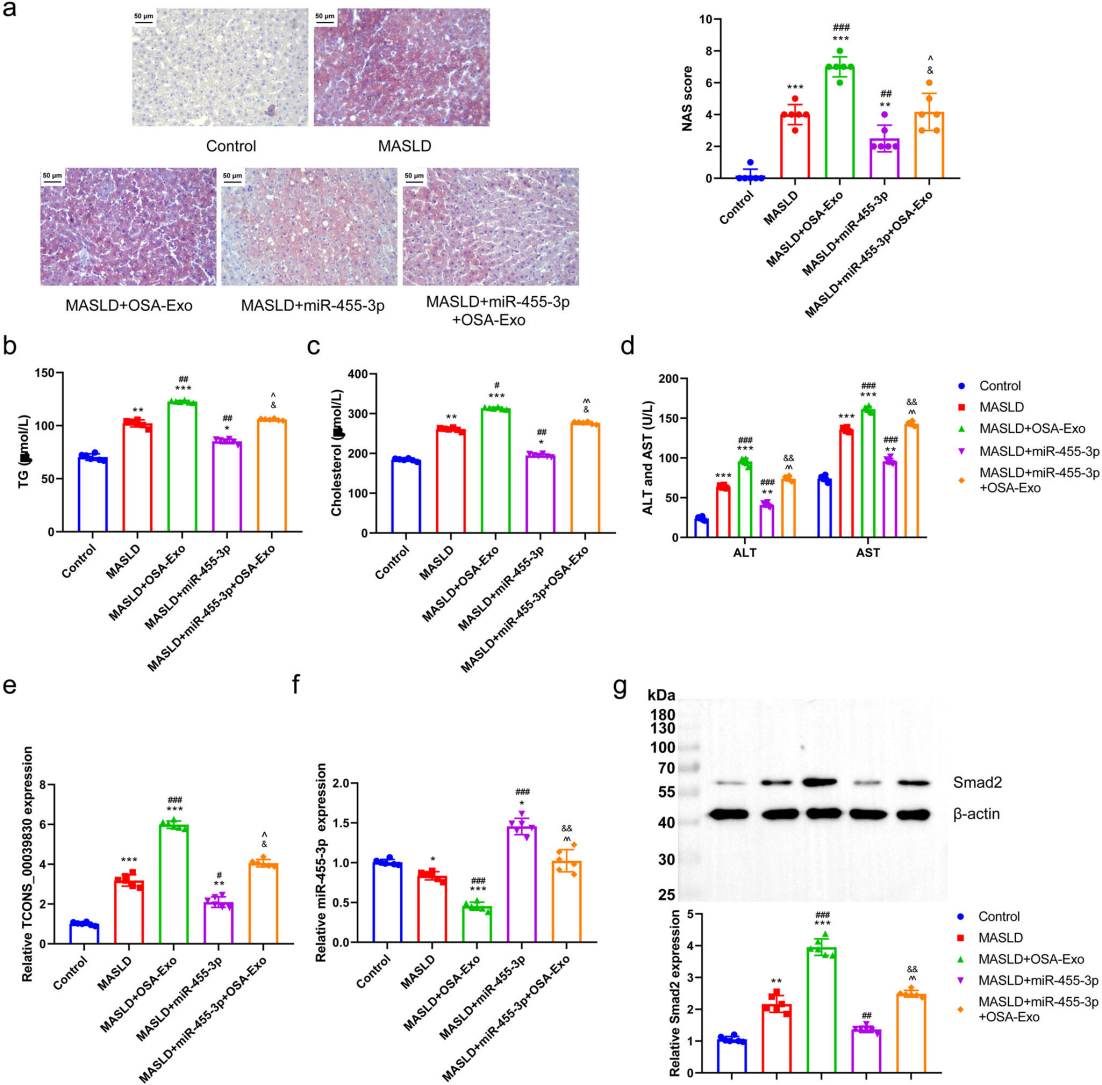
Exosome-delivered TCONS_00039830 regulates fat accumulation through miR-455-3p. **a** Fat accumulation in liver tissues detected by oil red O staining, as well as quantification result of NAS activity score to evaluate histological changes; **b** Triglyceride level of rat serum in Control, MASLD, MASLD + OSA-Exo, MASLD + miR-455-3p, and MASLD + miR-455-3p + OSA-Exo groups; **c** Cholesterol level of rat serum in Control, MASLD, MASLD + OSA-Exo, MASLD + miR-455-3p, and MASLD + miR-455-3p + OSA-Exo groups; **d** Aspartate aminotransferase (ALT) and alanine aminotransferase (AST) levels of rat serum in Control, MASLD, MASLD + OSA-Exo, MASLD + miR-455-3p, and MASLD +miR-455-3p+OSA-Exo groups; **e** The relative expression of TCONS-00039830 in liver tissues of rats in Control, MASLD, MASLD + OSA-Exo, MASLD + miR-455-3p, and MASLD + miR-455-3p + OSA-Exo groups; **f** The relative expression of miR-455-3p in liver tissues of rats in Control, MASLD, MASLD + OSA-Exo, MASLD + miR-455-3p, and MASLD + miR-455-3p + OSA-Exo groups; **g** The relative mRNA and protein expressions of Smad2 in liver tissues of rats in Control, MASLD, MASLD + OSA-Exo, MASLD + miR-455-3p, and MASLD + miR-455-3p + OSA-Exo groups. *n* = 6 rats/group; **P < 0.01, ***P < 0.001 versus Control group; ^#^*P* < 0.05, ^##^*P* < 0.01, ^###^*P* < 0.001 versus MASLD group; ^*P* < 0.05, ^^*P* < 0.01 versus MASLD + OSA-Exo group; ^&^*P* < 0.05, ^&&^*P* < 0.01 versus MASLD + miR-455-3p group. MASLD Metabolic Dysfunction-associated Steatotic Liver Disease, OSA Obstructive Sleep Apnoea. x̅ ± SD.

Fat accumulation and the expression of TCONS_00039830, miR-455-3p and *Smad2* were examined in the Control, MASLD, MASLD + OSA-Exo, MASLD + miR-455-3p and MASLD + miR-455-3p + OSA-Exo groups to verify the role of miR-455-3p in mediating the regulatory effects of exosome-mediated TCONS_00039830 on fat accumulation. Oil red O staining images revealed that miR-455-3p decreased the fat accumulation in the MASLD and MASLD + OSA-Exo groups. The MASLD + OSA-Exo group had the highest NAS followed by MASLD + miR-455-3p + OSA-Exo, MASLD, and MASLD + miR-455-3p groups (Fig. 9a). Additionally, miR-455-3p decreased the increased levels of TG, cholesterol, ALT and AST (Fig. 9b–d) and the high expression of TCONS_00039830, miR-455-3p and *Smad2* (Fig. 9e–g) in the MASLD and MASLD + OSA-Exo groups. Overall, these results suggest that exosome-mediated TCONS_00039830 targets miR-455-3p to regulate fat accumulation.

## Discussion

In this study, TCONS-00039830 and *Smad2* were upregulated and miR-455-3p was downregulated in rats with MASLD and those with MASLD and OSA. TCONS_00039830 overexpression aggravated and miR-455-3p overexpression ameliorated hepatic steatosis in the rat model. Furthermore, the accumulation of fat, TGs and cholesterol was higher in the FFA + Exo and FFA + OSA-Exo groups compared with that in the FFA and FFA + OSA groups. Exosomes derived from rats with OSA increased the expression of TCONS_00039830 and *Smad2* and decreased the expression of miR-455-3p. MiR-455-3p overexpression reversed the increased fat accumulation and upregulation of TCONS_00039830 and *Smad2*. Altogether, exosomes derived from rats with OSA promoted hepatic steatosis by increasing the activity of the TCONS_00039830/miR-455-3p/*Smad2* axis in MASLD.

Intermittent nocturnal hypoxia may contribute to the pathogenesis of MASLD by dysregulating liver inflammation^19^. This type of hypoxia regulates lipid biosynthetic pathways, hepatic steatosis, oxidative stress, liver inflammation and fibrosis^20^. RNA expression profiles for MASLD obtained using high-throughput sequencing, indicated that miR-455-3p is downregulated in MASLD^21^. Our findings also revealed that miR-455-3p is a co-differentially expressed miRNA between the MASLD + OSA and Control groups and between the MASLD + OSA and MASLD groups. TCONS-00039830 and *Smad2* were upregulated and miR-455-3p was downregulated in the MASLD and MASLD + OSA groups. MiR-455-3p is involved in various molecular functions and pathways associated with pro-inflammatory mechanisms and vascular remodelling^22^. Fei et al. reported that miR-455-3p participates in fat deposition by targeting CPT1A and DKK3^23^. In this study, the ceRNA network revealed that TCONS-00039830 and *Smad2* are located upstream and downstream of miR-455-3p, respectively. The liver is a critical regulator of lipid metabolism in humans^24^. MiR-455-3p can improve lipid metabolism in the liver by inhibiting the suppressor of cytokine signalling 3 through SREBP-1c^25,26^. Han et al. confirmed that the GATA3/miR-455-3p/*Smad2* cascade is involved in regulating muscle development in C2C12 myoblasts^27^. MiR-455-3p inhibited the activation of hepatic stellate cells by targeting the Hsp47/TGF-beta/Smad4 signalling pathway and was recommended as a promising therapeutic target for liver fibrosis^28^. Furthermore, the downregulation of TGFβ1-induced *Smad2/3* activation attenuated liver fibrosis and decreased morbidity and mortality among patients with chronic liver inflammation^29^. *Smad2* is upregulated in OSA^30^ and can protect against fibrosis by counteracting the TGF-β/Smad3 signalling^31^. Therefore, hepatic steatosis is worsened in MASLDMASLD with TCONS_00039830 overexpression and ameliorated in MASLD with miR-455-3p overexpression.

Furthermore, OSA increased MASLD-induced accumulation of fat, TGs and cholesterol. Swastik *et al*. showed that OSA is an important predictor of hepatic fibrosis in patients with MASLD^32^. However, the exact mechanism underlying the relationship between OSA and MASLD remains unclear. It is showed that circulating FFA and the sympathetic nervous system are upregulated in OSA^33^. In this study, accumulation of fat, TGs and cholesterol was higher in the FFA + Exo and FFA + OSA-Exo groups compared with that in the FFA and FFA + OSA groups, suggesting the mediating role of exosomes between OSA and MASLD.

Exosomes are derived from almost all types of cells in the body and contain multiple lipids, DNA, RNA, proteins and metabolites. Zhou et al. showed that brown adipose tissue-derived exosomes play a role in alleviating lipid accumulation in high-fat diet-fed mice^34^. Exosomes in patients with OSA may directly aggravate hepatic steatosis or act as a mediator to aggravate hepatic steatosis in MASLD. Exosomal miRNAs mediate intercellular communication, and prior author has reported the use as potential prognostic and diagnostic biomarkers in OSA^35^. Chaturvedi et al. demonstrated that exosomes mediate miR-455-induced downregulation of MMP-9 expression, which further alleviates myocardial fibrosis^36^. Shao et al. showed that mesenchymal stem cell-derived miR-455-3p-enriched exosomes isolated from the human umbilical cord can alleviate systemic disorders and improve the histology of the liver in patients with acute inflammatory liver injury^37^. Therefore, we hypothesised that exosomes may deliver TCONS_00039830, miR-455-3p and *Smad2* for modulating fat accumulation in hepatocytes.

Additionally, our findings indicate that exposure to adipocyte-derived exosomes isolated from rats with OSA increased the expression of TCONS_00039830 and *Smad2* and decreased the expression of miR-455-3p. Exosomes derived from rats with OSA exerted a greater effect on liver cells compared with those derived from normal Control adipocytes. The increased *Smad2* expression in MASLD and its role in hepatic fat accumulation have been widely reported^38^. *Smad2* is associated with miR-455-3p in various diseases, including Alzheimer’s disease and oesophageal squamous cell carcinoma^39,40^. In this study, dual-luciferase reporter assay validated the relationship among TCONS_00039830, miR-455-3p and Smad2. Upregulated TCONS_00039830 increased *Smad2* expression and decreased miR-455-3p expression, suggesting that TCONS_00039830 mediates the interaction between miR-455-3p and *Smad2* in MASLD. Further, exposure to exosomes derived from rats with OSA increased the expression of TCONS_00039830 and *Smad2*. These genes can contribute to the progression of hepatic steatosis in MASLD. The expression of TCONS_00039830 and *Smad2* was higher in the MASLD + OSA-Exo group compared with the MASLD group. Taken together, our findings reveal that exosomes derived from rats with OSA increase the risk of hepatic steatosis in MASLD by regulating the expression of TCONS_00039830/miR-455-3p/*Smad2* in hepatocytes.

However, the present study has some limitations. First, adipose tissue was used as the source of extracellular exosomes. Although adipose tissue is the key to MASLD and one of the main sources of extracellular vesicles, the influence of extracellular vesicles from other tissues should also be studied. Second, differentially expressed lncRNAs other than TCONS_00039830 are also found in exosomes in addition to mRNAs, proteins and lipids. Moreover, the biological functions of exosomes and the basic molecular mechanisms of the transport and function should be further explored. Third, OSA is caused by respiratory abnormalities, whereas the model in the present study was induced by exposure to reduced oxygen concentrations. Although the OSA modelling based on chronic intermittent hypoxia has been widely used in multiple studies^41–43^, a suitable model for OSA is needed to verify the significance of the TCONS_00039830/miR-455-3p/*Smad2* axis in OSA and MASLD.

Our study provides insights into the potential role of adipocyte-derived exosomes in the mechanism by which OSA aggravates MASLD. Exosomes derived from rats with OSA increased TCONS_00039830 expression in hepatocytes, thereby modulating the TCONS_00039830/miR-455-3p/*Smad2* axis. Our findings imply that treatment with miR-455-3p-derived exosomes or inhibition of hepatic expression of TCONS_00039830 represents a potential therapeutic approach for inhibiting the progression of MASLD. However, further studies are required to clarify the underlying mechanism by which the TCONS_00039830/miR-455-3p/*Smad2* axis is involved in MASLD development.

## Methods

### Establishing rat models of MASLD and MASLD + OSA

Male clean-grade Sprague–Dawley rats (*n* = 48; 200 ± 20 g) were purchased from Shanghai Slack Experimental Animal Co., Ltd. (Shanghai, China). The rats were housed in an environment with a relative humidity of 45–55% at 18–22 °C and allowed free access to water and food for one week. 12 rats were fed a standard chow diet (53% carbohydrates, 23% proteins and 5% fats; El-Nasr Company, Abu Zaabal, Egypt) during the second week and included in the healthy Control group. The remaining 36 rats were fed a high-fat diet consisting of 30% sucrose, 4% palmitic acid, 2% cholesterol and 0.5% cholic acid (Sigma-Aldrich, St Louis, MO, USA) and 30% lard stearin (El-Nasr Company, Abu Zaabal, Egypt) and included in the MASLD group. Rats in both groups were euthanised using 1% pentobarbital sodium after eight weeks, and their liver tissues were isolated, immersed in 10% buffered formalin and embedded in paraffin. The formalin-fixed liver tissues were stained with haematoxylin and eosin (H&E; Sigma-Aldrich) for histologic analysis. The tissue samples were initially incubated with haematoxylin for 10 min at 25 °C and then with 0.5% eosin for 3 min. Oil red O staining was performed to observe lipid droplets in the liver and verify the establishment of MASLD models.

Nonalcoholic fatty liver disease (NAFLD) activity score (NAS), which originally evaluated 14 histologic features, was calculated to analyse histologic changes^44^. Five features are independently associated with MASLD diagnosis, namely steatosis, hepatocellular ballooning, lobular inflammation, fibrosis and the absence of lipogranuloma. NAS (0–8) is determined by calculating the sum of three equal-weighted features: steatosis (0–3), lobular inflammation (0–3) and hepatocellular ballooning (0–2). NAS ≥ 5 correlates with MASLD, whereas NAS < 3 is not considered MASLD.

Chronic intermittent hypoxia (CIH) was used to construct an OSA model in this study. The induction principle of OSA-related diseases including MASLD was caused by chronic hypoxia caused by OSA^41–43^. OSA could directly cause a decrease in oxygen inhalation. Therefore, the hypoxia caused by OSA was simulated by directly controlling the inhaled concentration of oxygen, that was, CIH was used to simulate OSA. We kept 6 rats with MASLD in a glass chamber (30 × 20 × 20 in; BioSpherix, Redfield, NY, USA) to control the concentration of inhaled O_2_. The intermittent low O_2_ concentration was provided by keeping the average O_2_ concentration at 10 ± 1% for 60 s (hypoxia phase) and then raised to 20–21% for 30 s (normal phase). OSA models were constructed by maintaining the rats under intermittent low O_2_ concentration for 8 h/day (9:00 am to 5:00 pm) for 6 weeks^41–43^. The 24 rats of MASLD model were randomly divided into four groups, namely MASLD, MASLD + OSA-Exo, MASLD + miR-455-3p and MASLD + miR-455-3p + OSA-Exo groups.

All animal experiments were performed in accordance with the guidelines of the Animal Experiment Committee of Yan’an Hospital affiliated with Kunming Medical University. We have complied with all relevant ethical regulations for animal use.

### Exosome isolation and labelling

Epididymal white adipose tissue of rat was collected to isolate exosomes for further research. Firstly, the tissue was washed and cut into 1 mm^3^ pieces. Then, pieces were suspended using DMEM/F12 medium (Gibco, CA, 12500062) supplemented with 1% antibiotics and 10% fatal bovine serum (FBS). Notably, bovine exosomes were firstly depleted through overnight ultracentrifugation at 100,000 × *g*. Exosomes were extracted using the ExoQuick and ExoQuick-TC kit (System Biosciences, Mountain View, CA) according to the manufacturer’s instructions at 4 °C. Fresh rat serum/cell culture medium was mixed with the provided reagent to separate exosomes. The pellets were firstly collected and resuspended in PBS. Exosome suspension (8 μL) was added to carbon-coated 220 mesh copper grids, and exosomes were allowed to adhere for 2 min. Thereafter, 1% phosphotungstic acid (Sigma-Aldrich) was added and allowed to dry at 25 °C for 1 h. Finally, the exosomes were observed at an acceleration voltage of 70 kV using a transmission electron microscope (TEM; JEM 1010, JEOL, MA, USA)^45^. A particle size analyser (NanoSight LM10, Malvern, UK) was used to detect the particle size distribution of exosomes. Western blotting was performed to detect the expressions of exosome marker proteins (CD9, CD63 and Cyto C).

### Experimental design and transfection

30 rats were divided into five groups, namely Control, MASLD, MASLD + OSA-Exo, MASLD + miR-455-3p and MASLD + miR-455-3p + OSA-Exo groups. Rats in the Control group were housed at 25 °C under normal conditions and fed a normal diet. Rats with MASLD were intraperitoneally injected with 50 μg of exosomes isolated from rats with OSA once every 3 d in the MASLD + OSA-Exo group. In addition, 50 nM of miR-455-3p mimic (GenePharma, Shanghai, China) was injected into the tail vein of rats in the MASLD + miR-455-3p group, and the rats were maintained for 4 weeks. Rats in the MASLD + miR-455-3p + OSA-Exo group were simultaneously injected with both exosomes and miR-455-3p mimic. The rats were euthanised after 6 weeks, and their liver tissues were collected.

### RNA extraction

Total RNA was extracted from the liver tissues of three healthy rats, three rats with MASLD and three rats with MASLD and OSA using the TRIzol reagent (Invitrogen, Carlsbad, CA, USA) according to the manufacturer’s instructions. The concentration and integrity of the extracted RNA were estimated using a NanoDrop ND-1000 (NanoDrop, Wilmington, DE, USA) and a Bioanalyzer 2100 (Agilent, CA, USA) system, respectively. RNA was considered suitable for library construction when the RNA integrity number was >7.0. Ribosomal RNA was removed before library construction using the Epicentre Ribo-Zero Gold Kit (Illumina, San Diego, USA) according to the manufacturer’s instructions.

### Library construction and sequencing

An RNA library was constructed using the TruSeq Small RNA Sample Prep Kit (Illumina) according to the manufacturer’s instructions after quality and purity tests. RNA with poly (A) was purified using oligo (dT) magnetic beads and fragmented using the magnesium RNA fragmentation module (NEB, cat. e6150, USA). The fragmented RNA was reverse transcribed to cDNA using six random hexamer primers and a template of short fragments. Subsequently, second-strand cDNA was synthesised using a buffer, dNTPs, RNase H and DNA polymerase I.

The products were purified using AMPure XP beads, and cohesive ends were modified into blunt ends. Poly-adenylated caps were added at the 3′-ends using T4 DNA polymerase and Klenow DNA polymerase. The final sequencing library was prepared using PCR and sequenced on the Illumina Hiseq2000 platform (Illumina San Diego, CA, USA). The fastp tool was used to remove adaptor dimers, junk, repeats and reads containing >50% low-complexity regions.

### Analysis of differentially expressed genes

The classic Bayesian *t*-test was performed using the “limma” package to estimate differences in gene expression between two sets of samples. The Benjamini–Hochberg method was used to increase the reliability. MiRNAs with *p*-values of <0.05 were defined as differentially expressed miRNAs. StringTie was used to evaluate and quantify mRNAs and lncRNAs by calculating fragments per kilobase of exon per million mapped fragments [FPKM = total exon fragments/mapped reads (millions) × exon length (kB)]. CircRNAs were quantified by calculating spliced reads per billion mappings (SRPBM = number of back-spliced junction reads/number of mapped reads × 1,000,000,000). *P*-values were calculated using the parametric F-test in the R package “edge”^45^, and mRNAs, lncRNAs and circRNAs with *p*-values of <0.05 were considered differentially expressed mRNAs, lncRNAs and circRNAs, respectively.

### Functional analysis

A protein–protein interaction network of differentially expressed genes was constructed using the STRING database (v11.0), and interaction pairs were included if the interaction score was >0.4. Kyoto Encyclopaedia of Genes and Genomes (KEGG) pathway enrichment and Gene Ontology (GO) functional annotation analyses were performed using the Database for Annotation, Visualisation and Integrated Discovery (DAVID, v6.8) tool to investigate the potential functions of differentially expressed genes. Functional annotation in GO was based on three partially overlapping categories, namely molecular functions, biological processes and cellular components.

### Construction of a lncRNA–miRNA–mRNA network

The TargetScan (release 5.0: http://www.targetscan.org) and miRanda (http://www.microrna.org/microrna/home.do) tools were used to predict related target genes of miRNAs. MiRNA–mRNA and miRNA–lncRNA interactions were then integrated into a competitive endogenous RNA (ceRNA) network. Only upregulated lncRNAs and downregulated miRNAs were integrated with upregulated mRNAs, whereas only downregulated lncRNAs and upregulated miRNAs were integrated with downregulated mRNAs. LncRNAs in the ceRNA network that interacted with more than five miRNAs were considered key lncRNAs. Finally, the networks were visualised using Cytoscape (v3.6.0).

### Isolation of primary hepatocytes

Primary hepatocytes were isolated from the liver tissues of Control rats. Liver tissues were placed in a 150-mm petri dish containing sterile medium (1 g/L glucose, 15 mM HBSS and 1% BSA; Gibco, Carlsbad, CA, USA) and cut into small pieces using a scalpel blade. The tissues were digested using a mixture of collagenase and protease (Vitacyte, Indianapolis, IN, USA), filtered (pore size: 105 μm) and centrifuged at 70 × *g* for 3 min at 4 °C. Hepatocytes were pelleted through Percoll-based density gradient centrifugation (GE Healthcare, Marlborough, MA, USA). The final pellet was resuspended in DMEM/F12 medium (Gibco) supplemented with 10% foetal bovine serum (Gibco) and cultured at 37 °C in a 5% CO_2_ incubator.

### Construction of an MASLD model in vitro

Hepatocytes were randomly divided into three groups. One group was designed to assess the role of TCONS_00039830 in MASLD development, which included Control, FFA + si-TCONS_00039830, and si-TCONS_00039830. Primary hepatocytes were treated with 0.5 mmol/L FFA mixture (oleate: palmitate = 2:1; Sigma, Malaysia) for 24 h to construct an in vitro MASLD model followed the previous study^46^. Plasmids containing TCONS_00039830 siRNA were obtained from GenePharma. Approximately 5 × 10^5^ hepatocytes/well were seeded into 6-well plates and cultured overnight. The complete culture medium was replaced with a serum-free medium next day. SiRNA-TCONS-00039830 was then were transfected to hepatocytes after FFA treatment and hepatocytes using Lipofectamine® 3000 (Thermo Fisher Scientific, Inc., Cleveland, OH, USA) as FFA + si-TCONS_00039830 and si-TCONS_00039830, respectively. The serum-free medium was replaced with the complete culture medium after 6 h, and the efficiency of transfection was measured using reverse transcription quantitative polymerase chain reaction (RT–qPCR).

One group was designed to explore the potential regulation relationship between TCONS_00039830 and miR-455-3p, which included FFA, FFA + TCONS_00039830, FFA + miR-455-3p mimic, and FFA + TCONS_00039830 + miR-455-3p mimic. Plasmids containing TCONS_00039830-overexpression and miR-455-3p mimic were also obtained from GenePharma, and they were transferred into hepatocytes after FFA treatment as previously described.

The other group was designed to investigate the potential mediator role of exosomes in OSA induced hepatic steatosis, which included Control, FFA, FFA + OSA, FFA + Exo, FFA + OSA-Exo and FFA + OSA–si-TCONS_00039830-Exo. Hepatocytes were co-cultured with 20 μg/mL exosomes in the FFA + Exo group. Cobalt (II) chloride (CoCl_2_; Sigma Aldrich) is a well-known hypoxia-mimetic agent. Therefore, hepatocytes were treated with 200 μmol/L CoCl_2_ for 24 h to construct an intermittent hypoxia model in vitro, and Control cells were treated with only BSA^47^. Hepatocytes in the FFA + Exo, FFA + OSA-Exo and FFA + OSA-si-TCONS_00039830-Exo groups were co-incubated with exosomes isolated from Control rats, rats with OSA and rats with OSA and silenced TCONS_00039830, respectively.

### Dual-luciferase reporter assay

The 3′-UTR sequences of wild-type (wt) *Smad2* and wt-TCONS_00039830 were amplified to the downstream site of the pGL4 luciferase vector (Promega, Madison, WI, USA). The rapid site-directed mutagenesis kit (D0206; Beyotime, Shanghai, China) was used to generate the mutated (mut) SIRT3 mRNA 3′-UTR and mut-TCONS_00039830. 293T cells were seeded into 24-well plates at a density of 3 × 10^4^ cells/well. Approximately 1 μg of wt-*Smad2*/mut-*Smad2* or wt-TCONS_00039830/mut-TCONS_00039830 luciferase plasmid, 50 nM miR-455-3p mimic or miR-455-3p NC and 150 ng of Renilla luciferase plasmid (Beyotime) were transfected into 293T cells using the Lipofectamine^TM^2000 reagent after 24 h, and the cells were incubated at 37 °C for 36 h. A dual-luciferase reporter gene detection kit (Promega) was used to detect luciferase activity according to the manufacturer’s instructions. All data were normalised to Renilla luciferase activity.

### Oil red O and Bodipy staining

The liver tissue sections of rats were rinsed through reverse osmosis for 3 min and using 60% isopropanol for 30 s. The tissue sections were stained with oil red O (C0158M; Beyotime) for 20 min and subsequently rinsed with 60% isopropanol for 30 s. Thereafter, the tissue sections were rinsed for 1 min to remove residual isopropanol. Finally, the nucleus was stained with Mayer’s haematoxylin for 3 min. Bodipy staining was performed by initially staining the cells with 20 mg/mL Bodipy solution (D3922; Invitrogen) for 20 min in the dark at 37 °C. The cells were then washed three times with PBS. Afterwards, the cells were counterstained with 40, 6-diamidino-2-phenylindole (D8417; Sigma-Aldrich) for 2 min and again washed three times with PBS. Finally, the lipid droplets were observed using a microscope (Olympus, Tokyo, Japan).

### Estimation of triglyceride and cholesterol levels

The levels of triglycerides (TGs), total cholesterol, glutamic-oxaloacetic transaminase (AST), and glutamic-pyruvic transaminase (ALT) in liver tissues and cells were determined using enzymatic assay kits (E1025-105 and E1026-105, Applygen Technologies Inc., Beijing, China), ALT colorimetric assay kit, AST colorimetric assay kit, respectively (Jiancheng, Nanjing, China).

### RT–qPCR

Total RNA was extracted using TRIzol (Invitrogen). The concentration and quality of the extracted RNA were evaluated, and 600 ng RNA was reverse transcribed using the PrimeScript™ RT reagent Kit (Dalian TaKaRa Co., Ltd., Dalian, China). The expression levels of *Smad2*, TCONS-00039830 and miR-455-3p were measured using RT–qPCR on an ABI 7500 platform. β-actin was used as an internal Control for *Smad2*, and U6 was used as an internal Control for TCONS-00039830. The expression levels of these genes were calculated using the 2^−ΔΔCt^ method. The primers used for PCR were: β-actin-forward, 5′-TCCGTCGCCCGGTCCACACCC -3′ and β-actin -reverse, 5′-TCACCAACTGGGACGATATG -3′; U6-forward, 5′-CCCTTCGGGGACATCCGATA-3′ and U6-reverse, 5′-TTTGTGCGTGTCATCCTTGC-3′; *Smad2*-forward, 5′-TTCAGTTCCGCCTCCAATCG-3′ and *Smad2*-reverse, 5′-CTTACCAAAGGCAGCAAGCC-3′; miR-455-3p-forward, 5′-GCAGTCCATGGGCATATACAC-3′; TCONS-00039830-forward, 5′-AATCGGCTACGGATGACCTG-3′ and TCONS-00039830-reverse, 5′-GCTTAAGCTTACCTGTGGCA-3′.

### Western blotting

Total protein was extracted from cell lysates, separated on a 10% SDS– polyacrylamide gel, and transferred onto a polyvinylidene difluoride membrane. The membrane was blocked with 5% skimmed milk for 1 h at 25 °C and incubated with anti-CD9 (1:1000; ab307085), anti-CD63 (1:1000; Affinity Biosciences Cat# AF5117, RRID: AB_2837603), anti-Cyto C (1:5000; ab133504), anti-Smad2 (1:1000; ab280888) and anti-β-actin (1:2000; ab8227) antibodies for 2 h at 25 °C. Subsequently, the membrane was washed and incubated with a secondary antibody (1:5000; ab7097) for 1 h according to the manufacturer’s instructions. Finally, protein bands were visualised through enhanced chemiluminescence. The densitometric values of the bands were analysed using the ImageJ software and each band was evaluated thrice. β-actin was used as the internal control.

### Statistics and reproducibility

All data were expressed as the mean ± standard deviation (x̅ ± SD). One-way ANOVA with LSD post hoc testing was performed to compare individual group data and determine multiple comparison-adjusted p-values. All statistical analyses were performed using the SPSS Statistics software (v13.0) and GraphPad Prism 8.0. *N* = 3 biologically independent samples/animals/independent experiments. A *P*-value of <0.05 indicated statistically significant differences.

### Reporting summary

Further information on research design is available in the Nature Portfolio Reporting Summary linked to this article.

### Supplementary information

Description of Additional Supplementary Files

Supplementary Data 1

Supplementary Data 2

Supplementary Data 3

Supplementary Data 4

Supplementary Data 5

Supplementary Data 6

Reporting Summary

## Data Availability

All blot images presented in the figures are the original uncropped and unedited images. Source data for graphs and charts and all sequencing data can be found at https://zenodo.org/records/10686858. The numerical source data for Fig. 2a–f can be found at https://zenodo.org/records/10872667. All raw RNA-seq data were deposited in the Genome Sequence Archive (GSA) database under accession number CRA015629.
