# Supplementary material for: Adipocyte-derived exosomes from obstructive sleep apnoea rats aggravate MASLD by TCONS_00039830/miR-455-3p/Smad2 axis
Source: Commun Biol. 2024 Apr 23;7:492. doi: 10.1038/s42003-024-06171-z (PMC11039760; doi:10.1038/s42003-024-06171-z)
Supplement: Supplementary file 1 — Description of Additional Supplementary Files [file 42003_2024_6171_MOESM1_ESM.pdf]

Description of Additional Supplementary Files

File name: Supplementary Data 1
Description: Differentially expressed lncRNAs between the metabolic dysfunction-associated steatotic liver disease (MASLD) and Control groups.

File name: Supplementary Data 2
Description: Differentially expressed mRNAs between the metabolic dysfunction-associated steatotic liver disease (MASLD) and Control groups.

File name: Supplementary Data 3
Description: Differentially expressed lncRNAs between the metabolic dysfunction-associated steatotic liver disease (MASLD) + obstructive sleep apnoea (OSA) and Control groups.

File name: Supplementary Data 4
Description: Differentially expressed mRNAs between the metabolic dysfunction-associated steatotic liver disease (MASLD) + obstructive sleep apnoea (OSA) and Control groups.

File name: Supplementary Data 5
Description: Differentially expressed lncRNAs between the metabolic dysfunction-associated steatotic liver disease (MASLD) + obstructive sleep apnoea (OSA) and MASLD groups.

File name: Supplementary Data 6
Description: Differentially expressed mRNAs between the metabolic dysfunction-associated steatotic liver disease (MASLD) + obstructive sleep apnoea (OSA) and MASLD groups.
